# Sinus pericranii associated with syntelencephaly: a case report

**DOI:** 10.1186/s12883-022-02764-5

**Published:** 2022-08-25

**Authors:** Shuhei Fujino, Mikako Enokizono, Satoshi Ihara, Tatsuo Kono, Sahoko Miyama

**Affiliations:** 1 Department of Neurology, Tokyo Metropolitan Children’s Medical Center, 2-8-29 Musashidai, Fuchu, Tokyo 183-8561 Japan; 2 Department of Radiology, Tokyo Metropolitan Children’s Medical Center, 2-8-29 Musashidai, Fuchu, Tokyo 183-8561 Japan; 3 Department of Neurosurgery, Tokyo Metropolitan Children’s Medical Center, 2-8-29 Musashidai, Fuchu, Tokyo 183-8561 Japan

**Keywords:** Sinus pericranii, Syntelencephaly, Holoprosencephaly, Craniosynostosis, Tight posterior fossa, Case report

## Abstract

**Background:**

Sinus pericranii is a rare cranial venous malformation resulting in a subcutaneous mass due to abnormal communication between intracranial and subperiosteal/interperiosteal veins. To date, to the best of our knowledge, there are no reports of sinus pericranii associated with syntelencephaly, a subtype of lobar holoprosencephaly. We herein report a case of sinus pericranii associated with syntelencephaly. This report can provide us better understanding of the etiology of sinus pericranii, the potential risks, and the treatment options for these patients.

**Case presentation:**

A 2-year-4-month old female patient who received the diagnosis of syntelencephaly as a neonate presented with a subcutaneous mass in the parietal region. The mass was soft, nonpulsatile, 3 × 2 cm in size, and showed enlargement in the lying position. Color cranial Doppler ultrasound, head magnetic resonance imaging (MRI), and cerebral angiography revealed a dilated vessel passing through the parietal bone and forming a communication between the superior sagittal sinus and scalp veins. Based on these findings, sinus pericranii was diagnosed. The head MRI also showed coronal craniosynostosis, a tight posterior fossa. At age 2 years and 7 months, the patient underwent a transection of the sinus pericranii and the mass resolved without any complications or recurrences for more than 2.5 years to date.

**Conclusion:**

Sinus pericranii is a rare cranial and venous malformation sometimes accompanied by brain malformations or craniosynostosis that may become more apparent as the brain and skull develop. Since this condition can be complicated by intracranial hemorrhage and sinus thrombosis, early detection is necessary to determine the treatment options. Physicians should be alert to the possibility of this condition if they observe a soft cranial mass that appears to decrease in size in the sitting position and bulge in the lying position.

## Background

Sinus pericranii is a rare venous malformation presenting a subcutaneous venous mass resulting from abnormal communication between the intracranial and subperiosteal/interperiosteal veins [1]. Syntelencephaly, a subtype of lobar holoprosencephaly, characterized by poor separation of the posterior frontal and parietal lobes and corpus callosal hypoplasia/aplasia, accounts for 15%–17% of holoprosencephaly cases [2].

Cases of sinus pericranii associated with holoprosencephaly (including syntelencephaly), to our knowledge, thus far have not been reported. Since this condition can be complicated by intracranial hemorrhage and sinus thrombosis, early detection is necessary to determine the treatment options. For the better understanding of the rare condition of sinus pericranii, we herein report a case of sinus pericranii associated with syntelencephaly and discuss the treatment options, the potential risks for these patients, and the causal relationship between sinus pericranii and congenital malformations of the brain and/or skull.

## Case presentation

**Fig. 1:**
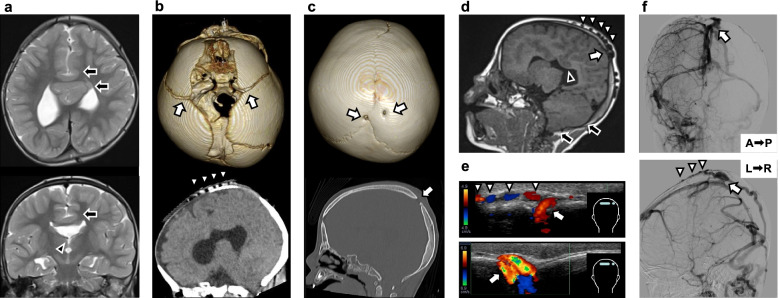
Imaging findings of syntelencephly, craniosynostosis, and sinus pericranii in the present case. **a** T2-weighted head MRI at age 2 years and 4 months. Abnormal midline connection between the cerebral hemispheres of the posterior frontal lobes and cingulate gyrus are shown (black arrow). The basal ganglia and thalamus are separated bilaterally (black arrow head). **b** Head computed tomography findings at age 4 months. The cranial sutures were not fused (white arrows). Tortuous and dilated subcutaneous veins were observed in the parietal region (white arrow heads). **c** Head computed tomography findings at age 2 years and 5 months. Two parietal skull bone defects (white arrows) and coronal craniosynostosis are visible. Digital impression on the skull was not evident. **d** T1-weighted head MRI at age 2 years and 4 months. Sinus pericranii (white arrow) via the parietal bone defect can be seen. The white arrowheads indicate distension of the subcutaneous veins in the parietal region. Callosal hypoplasia and ectopic gray matter around the lateral cerebral ventricles can also be observed. The black arrow indicates the tight posterior fossa and slight descent of the caudal part of the cerebellum toward the foramen magnum. Agenesis of the body of the corpus callosum and continuity of the cingulate gyrus across the midline are also shown. **e** Color cranial Doppler ultrasound findings at age 2 years and 5 months. Blood flow in two abnormal blood vessels (sinus pericranii) through the parietal bone defects to the extracranial veins can be seen (white arrow). The subcutaneous veins show distension (white arrow heads). **f** Cerebral angiography confirmed that the abnormal blood vessels formed a communication between the superior sagittal sinus and parietal diploic and subcutaneous veins (white arrows). No obvious thrombi, dural sinus abnormalities, or other vascular malformations were observed. The subcutaneous veins show distension (white arrow heads). L → R, left–right view (upper); A → P, anterior–posterior view (lower)

A 2-year-4-month old female patient who received the diagnosis of syntelencephaly as a neonate presented with a subcutaneous mass in the parietal region. Syntelencephaly was diagnosed based on head magnetic resonance imaging (MRI) findings, including an abnormal midline connection between the cerebral hemispheres of the posterior frontal lobes and cingulate gyrus, agenesis of the corpus callosum and septum pellucidum, and hypoplasia of the cerebral falx (Fig. 1a). Head computed tomography (CT) at 4 months of age showed tortuous and dilated subcutaneous veins in the parietal region (Fig. 1b). No evidence of craniosynostosis was detected at that time (Fig. 1b). She was affected by epilepsy, paraventricular heterotopia, congenital choanal atresia, and left facial cleft. She received antiepileptic drugs (carbamazepine, clonazepam, and zonisamide) and underwent plastic surgery for the facial cleft at Tokyo Metropolitan Children’s Medical Center. She had developmental delay; she began standing and walking with assistance at age 1 year and 7 months and walking without assistance at age 2 years and 4 months but has been unable to utter meaningful words.

She presented with a soft, nonpulsatile, subcutaneous mass 3 × 2 cm in size in the parietal region. The mass grew in size in the lying position and shrank in the sitting position. Head circumference at 2 years and 7 months was 48.4 cm, exhibiting age-appropriate development. Routine blood tests, including coagulation tests, were normal. Head computed tomography (CT) and MRI revealed abnormal vessels communicating the superior sagittal sinus with the distended, subcutaneous veins of the scalp via parietal bone defects along the sagittal suture and immediately next to it in the parietal region (Fig. 1c, d). The head CT also showed coronal craniosynostosis, which was not observed at 4 months of age (Fig. 1c). Digital impression on the skull was not evident on the head CT (Fig. 1c). Head MRI revealed tight posterior fossa and descent of the cerebellum and brain stem toward the foramen magnum (Fig. 1d). Color cranial Doppler ultrasound and cerebral angiography revealed two vessels communicating the superior sagittal sinus with the parietal diploic/subcutaneous veins (Fig. 1e, f). Based on these findings, sinus pericranii was diagnosed. Cerebral angiography disclosed no obvious thrombi, dural sinus abnormalities, or other vascular malformations, suggesting that transection of the sinus pericranii was not affecting intracranial venous perfusion. (Fig. 1f). Given the risk of serious hemorrhage in the event of a head injury, the decision to perform ligation and transection of the two sinus pericranii was made when the patient was aged 2 years and 7 months. The mass resolved postoperatively, and after more than 2.5 years to date, there have been no complications or recurrences.

## Discussion and conclusions

We reported a case of syntelencephaly, a rare brain morphological abnormality that was complicated by sinus pericranii, a rare venous malformation. To our knowledge, no cases of sinus pericranii associated with syntelencephaly have been reported, although several cases associated with craniosynostosis, as seen in this case, have been reported. In general, the factors contributing to sinus pericranii development include: 1) congenital, such as vascular and brain malformations; 2) acquired, such as increased intracranial and venous pressure; 3) physical, such as trauma; and 4) idiopathic [3].

In the present case, it is suggested that the congenital venous anomaly associated with syntelencephaly and the increased intracranial pressure due to craniosynostosis, impaired venous return due to tight posterior fossa, and increased physiological cerebral blood flow during infancy are complexly involved in the apparent sinus pericranii. Holoprosencephaly, including syntelencephaly, is often associated with various midline structural anomalies, including malformations of the cerebral sinuses, arteries, and veins, and the meningeal vessels [4]. Craniosynostosis is thought to occur in patients with inadequate brain growth or congenital brain malformation, suggesting a correlation between craniosynostosis and holoprosencephaly [5]. Stromeyer et al. reported that 7.2% of sinus pericranii cases were associated with craniosynostosis [6, 7]. In craniosynostosis, increased intracranial pressure due to brain growth is thought to cause cerebral venous blood flow to drain into the extracranial venous system, resulting in sinus pericranii formation [3, 6, 8]. In this case, a change in the intracranial pressure accompanying brain growth and coronal craniosynostosis may have resulted in sinus pericranii development. However, in the present case, tortuous and dilated subcutaneous veins in the parietal region were already observed at 4 months of age, before the cranial sutures were closed, suggesting that the congenital vascular malformation was primarily present. Therefore, the craniosynostosis probably did not directly cause formation of the sinus pericranii. However, the craniosynostosis may have contributed to exacerbation of the sinus pericranii. The tight posterior fossa and descent of the cerebellum observed in our case might have constricted venous return through the foramen magnum. In addition, the physiologic increase in cerebral blood flow during infancy may have contributed to the development of sinus pericranii. Cerebral blood flow physiologically increases until about age 5 [9]. In our case, the age of onset coincides with this period. Taken together, the sinus pericranii became apparent due to the congenital factor associated with syntelencephaly, and several other factors, including craniosynostosis, impaired venous return, and physiologic increases in cerebral blood flow in this case.

Sinus pericranii is often asymptomatic, as in this case; however, there are reports of intracranial and extracranial bleeding due to trauma or spontaneous rupture, posttraumatic air embolism, venous sinus thrombosis, and increased intracranial pressure due to venous congestion [10–12]. Since these complications are severe, examinations, including color cranial Doppler ultrasound and cerebral angiography, should be considered if a patient presents with a soft subcutaneous cranial mass that decreases in size while in the sitting position and bulges in the lying position, especially if brain and/or skull malformation are already known to exist, as in our case.

There is little evidence on which the treatment of sinus pericranii can be based. Transection may be performed to prevent the complications mentioned above and for aesthetic reasons [13]. However, surgical complications, such as bleeding, venous stasis, and air embolism, have also been reported in patients undergoing surgical treatment [14]. In a previous case series, patients who were treated with endovascular embolization had a favorable course; however, there were also concerns about the possibility of serious complications, such as epidermal necrolysis, deep venous thrombosis, and pulmonary embolization due to the procedure [15]. The previous study have been reported that surgical treatments on 7 cases of sinus pericranii associated with craniosynostosis were performed without any postoperative complications or recurrences [8]. In this case, surgery was chosen given the risk of serious hemorrhage and possibility of exacerbation of the lesion and a favorable outcome was achieved without complications or recurrence. The absence of dural sinus abnormalities on cerebral angiography was also a rationale for safe closure of the sinus pericranii. Aggressive treatment should be considered for cases of sinus pericranii accompanied by abnormalities of the brain and/or skull morphology.

As a general rule, surgery for craniosynostosis should be performed before surgery for sinus pericranii because sinus pericranii may serve as major draining veins of the intracranial venous blood in cases of craniosynostosis. Also, without reducing the intracranial pressure, sinus pericranii may recur. However, craniosynostosis repair was not performed in our case for the following reasons: 1) Sinus pericranii may have been present congenitally, and craniosynostosis was not considered a major cause of sinus pericranii. 2) No other vascular morphological abnormalities were found. 3) The head circumference developed well, and no skull deformities or symptoms of intracranial hypertension, such as headache, nausea, vomiting, or upgaze palsy, were observed [16]. In addition, this patient did not show cranial digital impression, which is indirect evidence of increased intracranial pressure. 4) The patient was over 2 years of age. Craniosynostosis repair is recommended within the first year of life because the brain volume increases significantly during this period [17]. In our center, craniosynostosis that develops after the age of 2 years is not an absolute indication for surgery if intracranial hypertension or cranial deformation is not observed. As a result, the patient had a good course with no recurrence over 2.5 years of follow-up. Nevertheless, surgery for craniosynostosis may be considered if the sinus pericranii recurs or intracranial hypertension occurs in the future.

Sinus pericranii is a rare cranial and venous malformation sometimes accompanied by brain malformations or craniosynostosis that may become more apparent as the brain and skull develop. Since sinus pericranii carries the risk of bleeding and thromboembolism, surgical treatment should be considered. The better understanding of sinus pericranii and causal relationship between sinus pericranii and congenital brain abnormalities is important for physicians to detect it early and to provide the treatment options for these patients. Physicians should be alert to the possibility of this condition if they observe a soft cranial mass that appears to decrease in size in the sitting position and bulge in the lying position. Early, neurosurgical intervention may be considered in such cases.

## Data Availability

The data that support the findings of this report are available from the corresponding author, SF, upon reasonable request.

## Abbreviations

CT: Computed tomography
MRI: Magnetic resonance imaging
